# Study of Radio Frequency Plasma Treatment of PVDF Film Using Ar, O_2_ and (Ar + O_2_) Gases for Improved Polypyrrole Adhesion

**DOI:** 10.3390/ma6083482

**Published:** 2013-08-13

**Authors:** Akif Kaynak, Tariq Mehmood, Xiujuan J. Dai, Kevin Magniez, Abbas Kouzani

**Affiliations:** 1 School of Engineering, Deakin University, Geelong, Victoria 3216, Australia; E-Mails: tmeh@deakin.edu.au (T.M.); abbas.kouzani@deakin.edu.au (A.K.); 2 Institute for Frontier Materials, Deakin University, Geelong, Victoria 3216, Australia; E-Mails: jane.dai@deakin.edu.au (J.D.); kevin.magniez@deakin.edu.au (K.M.)

**Keywords:** coatings, plasma treatment, surface modification, polypyrrole, interfacial bonding, conducting polymers, PVDF

## Abstract

Improvement of the binding of polypyrrole with PVDF (polyvinylidene fluoride) thin film using low pressure plasma was studied. The effects of various plasma gases *i.e.*, Ar, O_2_ and Ar + O_2_ gases on surface roughness, surface chemistry and hydrophilicity were noted. The topographical change of the PVDF film was observed by means of scanning electron microscopy and chemical changes by X-ray photoelectron spectroscopy, with adhesion of polypyrrole (PPy) by abrasion tests and sheet resistance measurements. Results showed that the increase in roughness and surface functionalization by oxygen functional groups contributed to improved adhesion and Ar + O_2_ plasma gave better adhesion.

## 1. Introduction

PVDF (polyvinylidene fluoride) is a semi-crystalline polymer in which each monomer has two dipole moments, one due to CF_2_ and the other due to CH_2_. The most common and most studied crystal structures are α and β phases [1,2]. The piezo-electric and pyro-electric properties of PVDF mainly depend on the β-phase content and its growth can be induced by several techniques, the most common being the mechanical stretching of the α-phase films at a suitable temperature [3]. Mechanical strains due to the bending, vibration or compression of the thin-film structure can be the source of the energy generation [4,5]. A PVDF sensor was integrated into an automotive seat cover fabric to obtain information about the car occupant [1]. Hutchison *et al.* [6] used platinum coating in between PVDF and polypyrrole (PPy) to get a stable connection in their actuator device. Choi *et al.* [7] used conductive fabric and PVDF to monitor cardiorespiratory signals employing the piezoelectric property of PVDF. The construction of a PVDF based energy harvesting device require electrodes on both sides of the PVDF film, these electrodes are connected to an electronic circuit to harvest electricity during motion. Different metal coatings can be used as electrode materials [8] and are brittle and uncomfortable to the skin. PPy coatings can be used instead of metals for this purpose due to ease of coating, comfort, and conformity [9]. Bhat *et al.* [10] synthesized a PVDF PPy composite film by electrochemical polymerization and Mansouri *et al.* [11] coated PPy onto PVDF microfiltration membranes by an *in situ* chemical polymerization method.

One of the perceived problems for PPy coating is poor adhesion due to low surface energy and the chemical nature of PVDF. Plasma can be used for the surface modification to improve subsequent coating deposition and bonding [12,13]. Exposing PVDF films to argon (Ar), hydrogen (H_2_), and oxygen (O_2_) remote plasma, introduced various functional groups [14]. Substitution of carbonyl or hydroxyl groups with fluorine atoms (de-fluorination) is central to the improvement of adhesion [14,15,16,17]. Duca *et al.* [17] investigated radio frequency (RF) Ar plasma treatment on PVDF. The water contact angle and fluorine to carbon (F/C) ratio was decreased, while surface roughness increased after the argon plasma treatment. Ar and O_2_ plasma treatments [18] incorporated oxygen onto the surfaces of PVDF. Our previous investigation [19] on a PET surface suggests that the improvement of adhesion of PPy was mainly due to incorporation of oxygen based functional groups on the surface after plasma treatment.

In this work, results of our investigations on PVDF under RF plasma for the improvement of adhesion of PPy are reported using Ar, O_2_ and (Ar + O_2_) gases. The surface morphology, wettability, chemical composition, electrical resistivity and abrasion resistance of PVDF were studied before and after each plasma treatment.

## 2. Experimental Procedure

### 2.1. Materials

The substrate was a PVDF thin film of 50 micron thickness. Films were immersed in acetone, ultrasonicated for 5 min, then washed with deionized water and dried at room temperature. Substrate materials were then conditioned under standard atmospheric pressure at 65% ± 2% relative humidity and 21 ± 1 °C for at least 24 h prior to further processing. Monomer (pyrrole), oxidant (ferric chloride FeCl_3_·6H_2_O) and dopant (para-toluene sulphonic acid mono hydrate, pTSA) reagent quality were purchased (Sigma-Aldrich, Melbourne, Australia).

### 2.2. Plasma Treatment

Plasma treatment of both PVDF thin films was performed in a custom built 13.5 MHz inductively coupled reactor [20]. The base pressure 1 × 10^−3^ mbar was achieved by use of a rotary pump. Ar plasma was used for the pre-treatment of the samples (100 W, 8 × 10^−2^ mbar, 30 s) to activate and clean the surface. Plasma power was kept at 100 W for all the gases. The gas pressure was set at 8 × 10^−2^ mbar for Ar and 8 × 10^−2^ mbar for both O_2_ and Ar + O_2_ samples. In case of Ar + O_2_, the composition of both gases was kept at 1:1 by volume. The plasma treatment time was fixed at 120 s for all the samples.

### 2.3. Contact Angle Measurement

Water contact angle (WCA) measurements were performed using a CAM100 WCA tester (KSV Instruments, Helsinki, Finland) and distilled water. The films were mounted on glass slides prior to measurement using double side adhesive tape. At least six measurements of each PVDF sample condition were taken to get an averaged WCA measurement. Attention Theta v4.1.9.8 (Biolin scientific, Stockholm, Sweden) software was used to calculate WCA from the captured images after 0.80 s of water droplet contact time. All the measurements were carried out within 10 min of plasma treatment.

### 2.4. Scanning Electron Microscopy

A Supra 55 VP (Carl Zeiss, Oberkochen, Germany) scanning electron microscope (SEM) was used to study the surface morphology of the PVDF thin film surface before and after plasma treatment. All the samples were gold coated before viewing in the SEM.

### 2.5. X-ray Photo Spectroscopy (XPS)

Spectra were obtained using a K-α X-ray photoelectron spectrometer (Thermo Fischer Scientific, Waltham, MA, USA) with monochromatic X-rays focused to a 400 µm spot size. Excessive charging of the samples was minimised using a flood gun. The binding energies of the samples were accurately established by charge shift; the binding energy peak of the F1s to 688.65 eV [14]. Survey spectra were obtained at pass energy of 100 eV while high resolution peak scans were performed at 20 eV pass energy. X-ray photoelectron spectroscopy (XPS) measurements were done within one week of plasma treatment.

### 2.6. Abrasion Resistance and Sheet Resistance

Circular PPy coated PVDF thin film samples were cut using a GSM (grams per square meter) cutter with 38 mm diameter and were abraded against a reference wool fabric under 9 kPa head weight in a Martindale abrasion tester (I.D.M instruments, Melbourne, Australia) for 0, 200 and 2000 abrasion cycles. Micrographs of the samples before and after abrasion were taken under the same lighting and exposure by a microscope (Olympus DP-71, Tokyo, Japan). Sheet resistance was measured using a SP4 four point probe head (Signatone, Tomkins Court Gilroy, CA, USA) and source meter (Keithley 2400, Solon, OH, USA) and employing the same pressure for all the samples [21]. Sheet resistance values were obtained from six different locations of each sample and later average values were calculated.

## 3. Results and Discussion

### 3.1. Contact Angle Measurement

**Figure 1:**
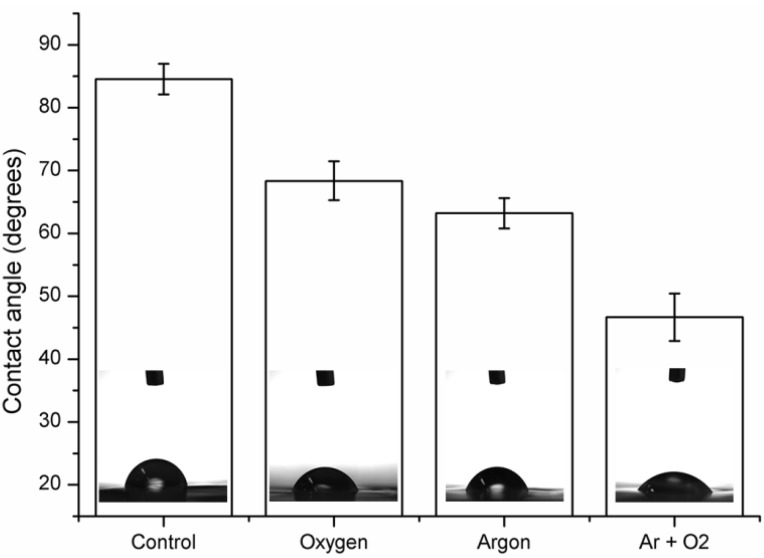
Water contact angle (WCA) of polyvinylidene fluoride (PVDF) thin film before and after Ar, O_2_ and (Ar + O_2_) plasma treatment.

Contact angle measurements show an improvement in the PVDF surface wettability after the plasma treatment. The average WCA value of the PVDF control sample was 84.55° and it reduced with plasma treatment irrespective of the gas used .WCA reduced to 68.36, 63.2 and 46.65 for Ar, O_2_ and Ar + O_2_ plasma samples respectively as shown in Figure 1. WCA is directly related to the surface energy, it is also dependent on surface morphology and chemical functional groups present on the surface.

### 3.2. Scanning Electron Microscopy

**Figure 2:**
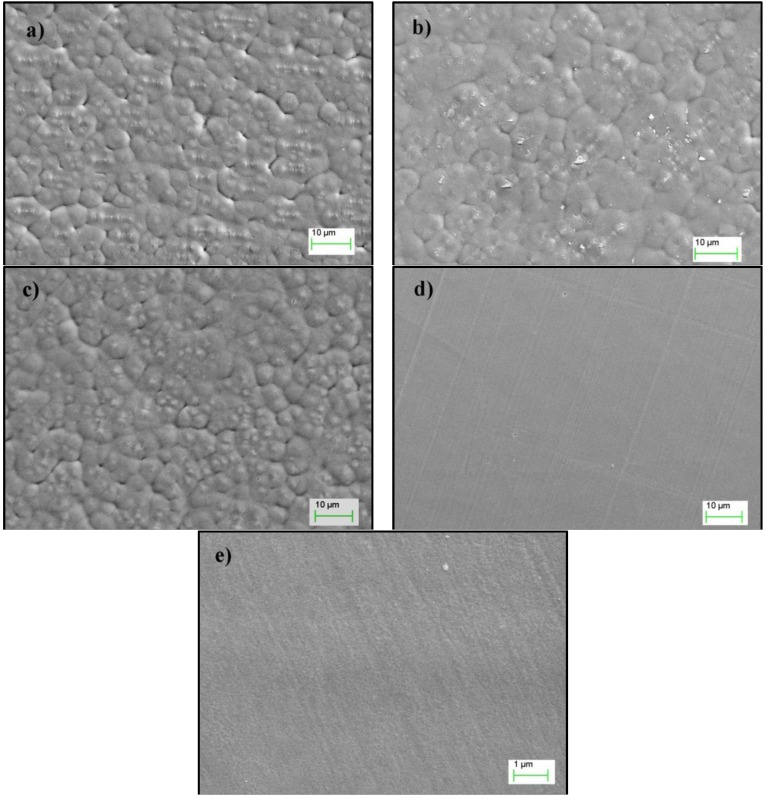
Scanning electron microscope (SEM) micrograph of polyvinylidene fluoride (PVDF): (**a**) control; (**b**) Ar plasma; (**c**) O_2_ plasma and (**d**,**e**) (Ar + O_2_) plasma.

Changes in the surface morphology are normally caused by ion bombardment and UV radiation in the plasma and depend on plasma gas. The SEM images in Figure 2a–e show the surface of the PVDF film before and after plasma treatment. The grainy features on the PVDF film surface were carried onward from the film manufacturing process.

There is no visible etching by Ar or O_2_ plasma whereas (Ar + O_2_) plasma treatment significantly altered the surface morphology (Figure 2d). A rougher surface was displayed as shown in higher resolution image (Figure 2e).

### 3.3. XPS Measurement

**Figure 3:**
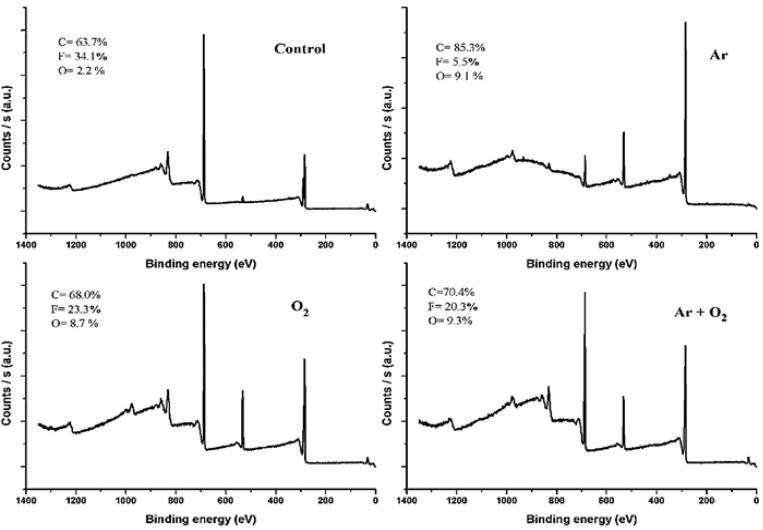
X-ray photoelectron spectroscopy (XPS) survey scan of polyvinylidene fluoride (PVDF) film before and after Ar, O_2_ and Ar + O_2_ plasma treatment.

XPS measurements were done for control, Ar, O_2_ and (Ar + O_2_) plasma treated PVDF samples. Carbon, fluorine and oxygen were detected in all the samples as shown in the overall XPS scan in Figure 3 and Table 1. Plasma treatment lowered the fluorine content, however oxygen was increased. There is a large difference in bonding energy between C–F and C–H bonds. The bond energy for F–F bonds is only 1.6 eV, that for H–F bonds 5.85 eV, and that for C–F bonds 4.42 eV [14]. High resolution C1s scans were performed to study the presence of different functional groups on the PVDF surface. C1s peaks were curve fitted to calculate the relative concentration of present functional groups. Three sub peaks C1s peak A, C1s peak B and C1s peak C at 286.4, 291.4 and 288 eV respectively were found. Peak A is assigned to CH_2_–CF_2_, CHF–CH_2_–CHF and O–CH_2_ groups. Peak B is assigned to CF_2_–CH_2_, while peak C is assigned to CH_2_–CHF–CH_2_, CH_2_–CHF–CHF, O–CH_2_–CF_2_ [22]. O 1s peak at 533.7 eV is assigned to C–O [23], while F1s peak at 688.65 eV to C–F bond.

**Table 1:** Atomic composition of untreated and plasma treated polyvinylidene fluoride (PVDF) film samples.

Sample	C1s Peak A (286.4)	C1s Peak B (291.4)	C1s Peak C (288)	O1s Peak A (533.7)	F1s Peak A (688.65)	C	O	F	O/C	F/C
Control	29.21	16.8	17.7	2.2	34.14	63.7	2.16	34.1	0.03	0.54
Ar	73.42	1.89	10	9.2	5.48	85.3	9.15	5.48	0.11	0.06
O_2_	51.54	7.99	8.46	8.7	23.31	68.0	8.7	23.3	0.13	0.34
Ar + O_2_	52.14	9.4	8.85	9.3	20.31	70.4	9.31	20.3	0.13	0.29

The untreated PVDF surface showed two main peaks at 291.4 eV (due to CF_2_) and 286.4 eV (due to CH_2_) components [11,24]. Ar plasma was the most effective in lowering the C1s peak B from 16.8 to 1.89, while O_2_ and (Ar + O_2_) resulted in moderate levels of de-fluorination (Table 1). The fluorine to carbon ratio (F/C) decreased from 0.54 to 0.06 for Ar and 0.34, 0.29 for O_2_ and (Ar + O_2_) respectively. Ar plasma also increased the surface oxygen content from 2.16% to 9.15%, however two other plasma gases achieved similar results for oxygen incorporation. The oxygen to carbon ratio (O/C) rose to 0.13 for O_2_ and (Ar + O_2_) plasma from 0.03 for the control sample. The O/C value was 0.11 for Ar plasma. It is interesting to note the incorporation of oxygen based functional groups into the argon plasma. The oxygen may arise from the following sources; (i) an impurity in Ar gas [25]; (ii) exposure to air after taking out of the plasma chamber before XPS measurement [17].

**Figure 4:**
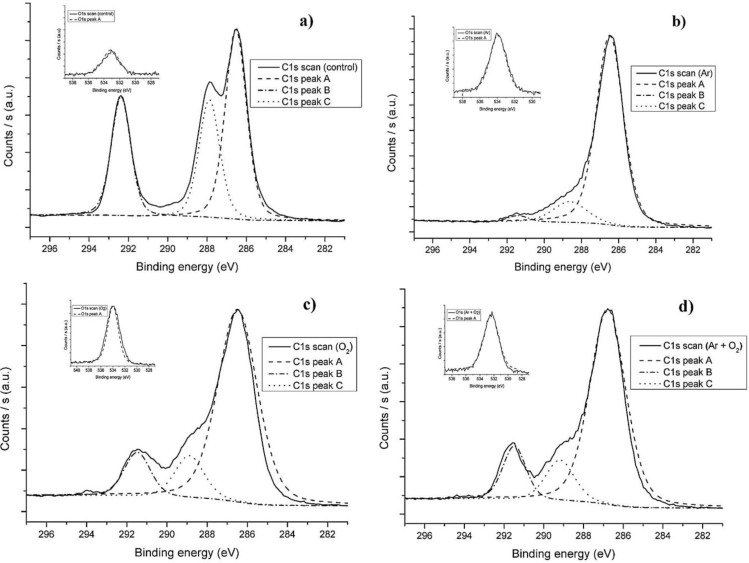
C1s scan spectra of polyvinylidene fluoride (PVDF). (**a**) Control; (**b**) Ar plasma; (**c**) O_2_ plasma and (**d**) (Ar + O_2_) plasma.

The C1s spectra of PVDF films before and after RF plasma show that plasma treatment causes de-fluorination and the incorporation of oxygen into the polymer surface (Figure 4). The C1s scans and overall composition (Table 1) indicate that CF_2_ carbons were modified into CHF and CH_2_ carbons and O–C carbons (O–CH_2_ and O–CHF) during plasma exposure as evident by the increase of oxygen content and decrease in C1s Peak A. Increase in hydrophilicity is an important indication of incorporation of oxygen based functional groups. Although Ar plasma resulted in a higher degree of de-fluorination, it did not result in lowering of WCA.

### 3.4. Abrasion Resistance and Sheet Resistance

**Figure 5:**
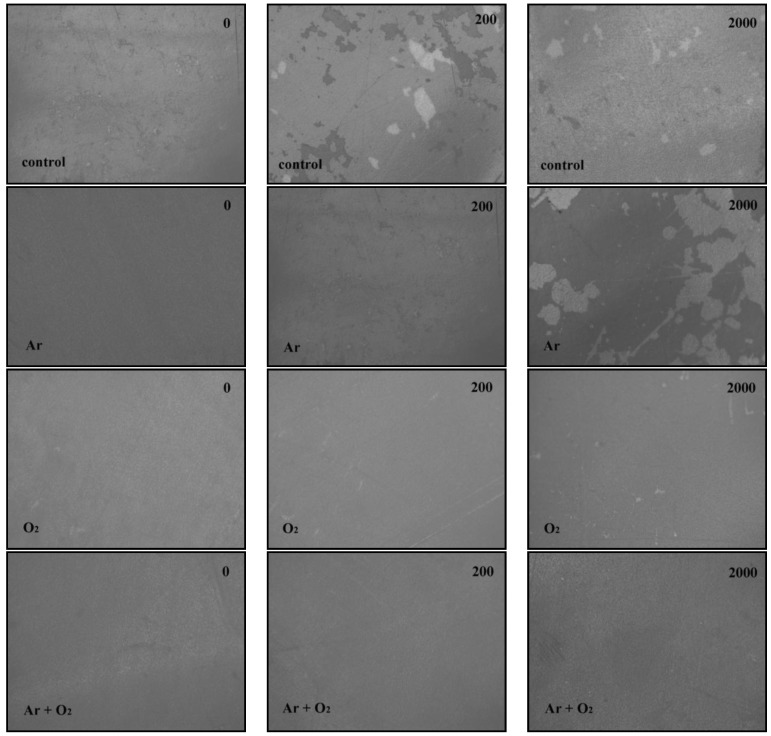
Polypyrrole (PPy) coated polyvinylidene fluoride (PVDF) film sample after 0, 200 and 2000 abrasion cycles in a Martindale abrasion tester for control, Ar, O_2_ and (Ar + O_2_) samples; magnification 10×.

PPy coated PVDF film samples abraded by the Martindale abrasion tester were photographed by a microscope (Olympus DP71, Tokyo, Japan) after 0, 200 and 2000 cycles of abrasion (Figure 5). The magnification was set at 10 times for all the images. Some of the coating of the control sample was missing even before the abrasion test due to weak interfacial bonding and nearly all the coating faded from the control sample after 200 and 2000 cycles of abrasion leaving the surface non-conductive.

Plasma treated samples clearly showed better abrasion resistance. Although the Ar plasma sample showed some flaking off after 2000 cycles, it was still significantly better than the control sample. On the other hand the O_2_ and (Ar + O_2_) plasma treated samples had strong binding as there was only a small visible coating loss. The effectiveness of (Ar + O_2_) plasma treatments is clearly evident, as it showed better coating fastness than use of these gases alone. This is due to de-fluorination and incorporation of oxygen based functional groups during plasma treatment [14]. The results showed that Ar plasma resulted in de-fluorination even before abrasion tests. We do not have evidence whether the surface morphology had any variation along with the de-flourination. An AFM investigation of this would be worthwhile in a future study.

**Figure 6:**
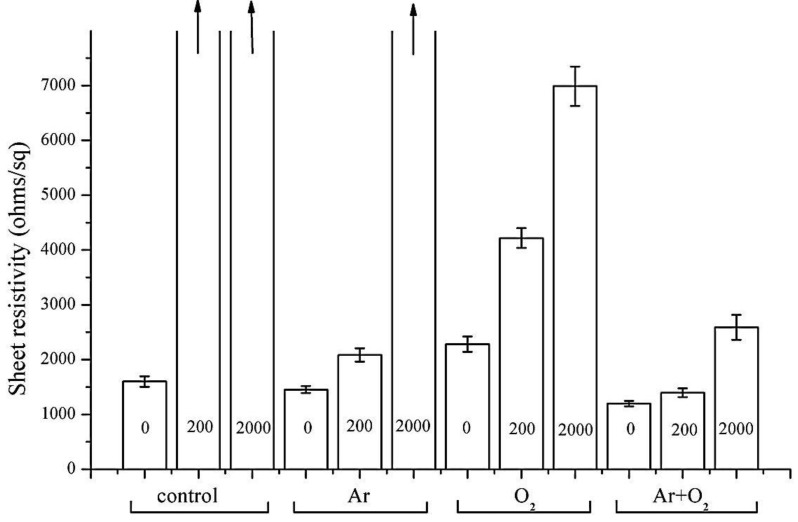
Sheet resistance of polypyrrole (PPy) coated polyvinylidene fluoride (PVDF) samples; control, Ar, O_2_ and (Ar + O_2_).

The Sheet resistance (R_s_) results after 0, 200 and 2000 abrasion cycles are presented in Figure 6. Ar and (Ar + O_2_) plasma gases lowered the R_s_ values of the PPy coated PVDF films compared to the control sample (1600 ohms/sq), while O_2_ plasma treatment resulted in a small increase in initial R_s_ values which may be due to O_2_ being less affected in de-fluorination compared to Ar and (Ar + O_2_). The R_s_ values could not be calculated for control samples with 200 and 2000 abrasion cycles. The lowest R_s_ values were noted for (Ar + O_2_) gas at 1197 ohms/sq for 120 s plasma treatment, which increased to 2589 ohms/sq after 2000 abrasion cycles. The R_s_ value of the Ar plasma sample before abrasion was 1452; it rose to 2083 and 66,523 ohms/sq after 200 and 2000 abrasion cycles respectively. Similarly, R_s_ values for O_2_ plasma before abrasion were 2280; they rose to 4218 and 6989 ohms/sq after 200 and 2000 abrasion cycles respectively. The improved sheet resistance as a result of the Ar + O_2_ plasma treatment may be attributed to the advantages of both the argon plasma (effective on defluorination) and the oxygen plasma (effective on dehydration and introduction of oxygen functional groups) to achieve improved bonding, hence lower resistivity.

The sheet resistance data of PPy coated PVDF samples in Figure 6 and photographs of abraded samples in Figure 5 agree with each other and all confirm the order of plasma gas effectiveness as (Ar + O_2_) > O_2_ > Ar. Contact angle measurements show an improvement in the PVDF surface wettability after the plasma treatment. The decrease of the F/C ratio and water contact angle prove that both chemical and physical effects play an important role in the adhesion.

It is interesting to note that control, Ar and O_2_ plasma treated PVDF samples had a grainy surface morphology which did not lower the R_s_ value as much as the (Ar + O_2_) plasma treated surface having a flat yet nano-sized surface roughness, a similar trend was observed for the bonding strength. Both XPS and surface imaging indicates that incorporation of oxygen functional groups and nano sized surface roughness are important for coating fastness. This was confirmed by the connection of abrasion resistance imaging, sheet resistance measurement and the chemical composition data in Table 1. Similar results were obtained in our previous work on polyester fabric [19].

The treatment of PVDF using oxygen, argon and hydrogen plasmas were reported in a similar study by Park *et al.* [26], where they reported using remote plasma treatment in which the sample was situated in the after-glow area and the authors focussed on the individual effects of the above mentioned plasma gases. Whereas, in our experiments the sample was placed inside the plasma source and the effects of argon (inert gas), oxygen (reactive gas) and the combination argon + oxygen plasma were used to enable a better understanding of the role of argon and oxygen plasma and the effect of the combination. Overall, results showed that the most effective treatment was the argon + oxygen combination, followed by oxygen and argon. Our results agree with that of Park *et al.* [26] in the contact angle data for oxygen and argon plasma, that is, argon plasma treatment was more effective than oxygen in hydrophilicity manifesting as a decrease in contact angle. Similarly, oxygen plasma was not very effective in defluorination, supporting the earlier study by Park *et al.* [26]. In this study we were able to use the advantages of both argon plasma (effective on defluorination) and oxygen plasma (effective on dehydration and introduction of oxygen functional group) to achieve improved bonding. In other words, the improved bonding of the combination may be attributed to the combined effect. Also the argon plasma produced more reactive sites on the substrate surface, which would enhance the binding of functional groups.

## 4. Conclusions

We have studied the improvement of binding of polypyrrole with a PVDF thin film using low pressure plasma. Ar, O_2_ and Ar + O_2_ gases were used in the plasma treatment. The abrasion data suggest adequate strength of bonding with the PVDF surface after plasma treatment. The plasma induced increase in hydrophilicity and surface functionalization with oxygen based groups contributed to the improved coating adhesion and fastness. These results clearly show the effectiveness of Ar + O_2_ plasma in lowering the resistivity and improving the binding strength of the PPy coating.

## References

[1] Drean E., Schacher L., Adolphe D., Bauer F., Leonhardt S., Falck T., Mähönen P. (2007). Smart Textiles for Automotive: Application to Airbag Development. 4th International Workshop on Wearable and Implantable Body Sensor Networks (BSN 2007).

[2] Moussaif N., Pagnoulle C., Riga J., Jérôme R. (2000). XPS analysis of the PC/PVDF interface modified by PMMA. Location of the PMMA at the interface. Polymer.

[3] Sencadas V., Gregorio R., Lanceros-Méndez S. (2009). α to β phase transformation and microestructural changes of PVDF films induced by uniaxial stretch. J. Macromol. Sci. Part B Phys..

[4] Chang J., Dommer M., Chang C., Lin L. (2012). Piezoelectric nanofibers for energy scavenging applications. Nano Energy.

[5] Kim H., Kim J.-H., Kim J. (2011). A review of piezoelectric energy harvesting based on vibration. Int. J. Precis. Eng. Manuf..

[6] Hutchison A.S., Lewis T.W., Moulton S.E., Spinks G.M., Wallace G.G. (2000). Development of polypyrrole-based electromechanical actuators. Synth. Met..

[7] Choi S., Jiang Z. (2006). A novel wearable sensor device with conductive fabric and PVDF film for monitoring cardiorespiratory signals. Sens. Actuators A Phys..

[8] Pascu M., Debarnot D., Durand S., Poncin-Epaillard F. (2005). Surface Modification of PVDF by Microwave Plasma Treatment for Electroless Metallization. Plasma Processes and Polymer.

[9] Wallace G.G., Campbell T.E., Innis P.C. (2007). Putting function into fashion: Organic conducting polymer fibres and textiles. Fibers Polym..

[10] Bhat N.V., Geetha P., Pawde S., Nallathambi R. (1995). Preparation of poly(vinylidene fluoride)–polypyrrole composite films by electrochemical synthesis and their properties. J. Appl. Polym. Sci..

[11] Mansouri J., Burford R.P. (1997). Characterization of PVDF-PPy composite membranes. Polymer.

[12] Sun J., Yao L., Gao Z., Peng S., Wang C., Qiu Y. (2010). Surface modification of PET films by atmospheric pressure plasma-induced acrylic acid inverse emulsion graft polymerization. Surf. Coat. Technol..

[13] Keller M., Ritter A., Reimann P., Thommen V., Fischer A., Hegemann D. (2005). Comparative study of plasma-induced and wet-chemical cleaning of synthetic fibers. Surf. Coat. Technol..

[14] Park Y.W., Inagaki N. (2004). A new approach for selective surface modification of fluoropolymers by remote plasmas. J. Appl. Polym. Sci..

[15] Yamada Y., Yamada T., Tasaka S., Inagaki N. (1996). Surface modification of poly(tetrafluoroethylene) by remote hydrogen plasma. Macromolecules.

[16] Crowe R., Badyal J.P.S. (1991). Surface modification of poly(vinylidene difluoride)(PVDF) by LiOH. J. Chem. Soc. Chem. Commun..

[17] Duca M.D., Plosceanu C.L., Pop T. (1998). Surface modifications of polyvinylidene fluoride (PVDF) under rf Ar plasma. Polym. Degrad. Stab..

[18] Momose Y., Noguchi M., Okazaki S. (1989). Ar, O_2_ and CF_4_ plasma treatment of poly-(vinylidene fluoride), polyimide and polyamidoimide and its relationship to wettability. Nucl. Instrum. Methods Phys. Res. Sect. B Beam Interact. Mater. At..

[19] Mehmood T., Dai X.J., Kaynak A., Kouzani A. (2012). Improved bonding and conductivity of polypyrrole on polyester by gaseous plasma treatment. Plasma Process. Polym..

[20] Dai X.J., Chen Y., Chen Z., Lamb P.R., Li L.H., du Plessis J., McCulloch D.G., Wang X. (2011). Controlled surface modification of boron nitride nanotubes. Nanotechnology.

[21] Bautista K. Four-Point Probe Operation. http://www.utdallas.edu/research/cleanroom/manuals/documents/4pointFinal.pdf.

[22] Vandencasteele N., Reniers F. (2010). Plasma-modified polymer surfaces: Characterization using XPS. Relat. Phenom..

[23] Pawde S.M., Deshmukh K. (2009). Surface characterization of air plasma treated poly vinylidene fluoride and poly methyl methacrylate films. Polym. Eng. Sci..

[24] Bodenes L., Naturel R., Martinez H., Dedryvère R., Menetrier M., Croguennec L., Pérès J.-P., Tessier C., Fischer F. (2013). Lithium secondary batteries working at very high temperature: Capacity fade and understanding of aging mechanisms. J. Power Sources.

[25] Dai X., Hamberger S., Bean R. (1995). Reactive plasma species in the modification of wool fibre. Aust. J. Phys..

[26] Park Y.W., Inagaki N. (2003). Surface modification of poly(vinylidene fluoride) film by remote Ar, H_2_, and O_2_ plasmas. Polymer.

